# Validation of the Spanish Version of the Yale Food Addiction Scale 2.0 (YFAS 2.0) and Clinical Correlates in a Sample of Eating Disorder, Gambling Disorder, and Healthy Control Participants

**DOI:** 10.3389/fpsyt.2018.00208

**Published:** 2018-05-25

**Authors:** Roser Granero, Susana Jiménez-Murcia, Ashley N. Gearhardt, Zaida Agüera, Neus Aymamí, Mónica Gómez-Peña, María Lozano-Madrid, Núria Mallorquí-Bagué, Gemma Mestre-Bach, Maria I. Neto-Antao, Nadine Riesco, Isabel Sánchez, Trevor Steward, Carles Soriano-Mas, Cristina Vintró-Alcaraz, José M. Menchón, Felipe F. Casanueva, Carlos Diéguez, Fernando Fernández-Aranda

**Affiliations:** ^1^Ciber Fisiopatología Obesidad y Nutrición, Instituto Salud Carlos III, Barcelona, Spain; ^2^Departament de Psicobiologia i Metodologia, Universitat Autònoma de Barcelona, Barcelona, Spain; ^3^Department of Psychiatry, University Hospital of Bellvitge-IDIBELL, Barcelona, Spain; ^4^Department of Clinical Sciences, School of Medicine and Health Sciences, University of Barcelona, Barcelona, Spain; ^5^Department of Psychology, University of Michigan, Ann Arbor, MI, United States; ^6^CIBER Salud Mental Instituto Salud Carlos III, Barcelona, Spain; ^7^Laboratory of Molecular and Cellular Endocrinology, Research Area Complejo Hospitalario Universitario de Santiago de Compostela, A Coruña, Spain; ^8^Department of Physiology, CIMUS University of Santiago de Compostela-Instituto de Investigación Sanitaria, Santiago de Compostela, Spain

**Keywords:** eating disorders, food addiction, gambling disorder, psychometric properties, validation, YFAS 2.0

## Abstract

**Aims:** Due to the increasing evidence of shared vulnerabilities between addictive behaviors and excessive food intake, the concept of food addiction in specific clinical populations has become a topic of scientific interest. The aim of this study was to validate the Yale Food Addiction Scale (YFAS) 2.0 in a Spanish sample. We also sought to explore food addiction and its clinical correlates in eating disorder (ED) and gambling disorder (GD) patients.

**Methods:** The sample included 301 clinical cases (135 ED and 166 GD), diagnosed according to DSM-5 criteria, and 152 healthy controls (HC) recruited from the general population.

**Results:** Food addiction was more prevalent in patients with ED, than in patients with GD and HC (77.8, 7.8, and 3.3%, respectively). Food addiction severity was associated with higher BMI, psychopathology and specific personality traits, such as higher harm avoidance, and lower self-directedness. The psychometrical properties of the Spanish version of the YFAS 2.0 were excellent with good convergent validity. Moreover, it obtained good accuracy in discriminating between diagnostic subtypes.

**Conclusions:** Our results provide empirical support for the use of the Spanish YFAS 2.0 as a reliable and valid tool to assess food addiction among several clinical populations (namely ED and GD). The prevalence of food addiction is heterogeneous between disorders. Common risk factors such as high levels of psychopathology and low self-directedness appear to be present in individuals with food addiction.

## Introduction

Similarities in the biological and psychological factors involved in substance abuse and pathological overeating have led some researchers to postulate that addictive processes may underlie excess food consumption (1–3). Studies have shown that the neural reward centers triggered by addictive substances (such as alcohol or drugs), or behavioral addictions, can also be activated by highly palatable food (4–11). Similar to drugs of abuse, the intake of macronutrient-rich foods may increase extracelluar dopamine in neural regions implicated in reward and motivation, thereby increasing the likelihood of reward-driven eating in the absence of homeostatic need (12–15). Likewise, there is evidence that repeated exposure to sugar-sweetened beverages can lead to frontostriatal adaptations and behavioral disinhibition (16). Patients with substance use disorders and behavioral addictions also report symptoms that are also often found in the context of eating disorders (EDs) or in individuals with excess weight. For example, some individuals report that they compulsively consume highly palatable foods despite trying to cut down on food intake or that they experience loss of control once they begin eating certain foods (17). Strong cravings for foods despite feeling full and spending a disproportionate amount of time to obtain food are also common occurrences in some ED patient populations (18, 19). Most importantly, people who present these symptoms continue overeating even though the consequences of excess food intake may cause significant functional impairment in the physical, personal and/or social domains of their life (20). These behaviors can all be considered within the context of the impulsive/compulsive spectrum (21), though few studies to date have placed food addiction within this model.

The Yale Food Addiction Scale (YFAS) (22) was the first validated instrument to measure addictive-like eating behavior, commonly referred to as food addiction. This instrument was based on the Diagnostic and Statistical Manual of Mental Disorders (DSM-IV-TR) (23) criteria for substance dependence and was adapted to the context of food consumption. Multiple studies using this questionnaire have verified its psychometric soundness and validity, and empirical data have linked high symptom count on the YFAS to: (a) eating related problems, such as obesity, binge eating disorders, food cravings and poor response to bariatric surgery (18, 24–27); and (b) altered neural response (28–30).

The prevalence of food addiction based on YFAS criteria has been found to range from 11 to 40%, depending on multiple factors such as the sample origin, participants' age (higher prevalence for middle-aged and older samples), sex (higher incidence in women), or lower socioeconomic level (18, 31–34). In addition to food addiction being associated with obesity and binge eating disorder (28, 35, 36), food addiction has also been observed in some normal-weight individuals who exhibit bulimic behavior (37). Levels of food addiction symptomatology appear to respond to psychological interventions, with one study finding that food addiction levels reduced after a short-term intervention in patients with bulimia nervosa (38). Interestingly, recent research has also linked food addiction symptomatology to gambling disorder (GD), suggesting that these two conditions may share common risk factors (19, 39, 40). Still, there is lack of consensus within the scientific community and among clinicians regarding the proper operational definition of food addiction (41–44). Some researchers have posited that “eating addiction” rather than food addiction might be a more accurate designation due to the fact that evidence regarding the addictive properties of specific foods is scarce (45).

The YFAS 2.0 was recently developed (46) to coincide with the new DSM-5 substance-related and addictive disorders (SRAD) criteria (47). The objective of these SRAD criteria is to assess a recurrent pattern of abusive consumption that leads to clinically significant distress. It includes core symptoms such as cognitive distortions, craving, abstinence and tolerance (47). In order to maintain consistency with the DSM-5 model of addiction and to make certain that the YFAS 2.0 reflected these changes, the updated scale added items regarding craving, merged abuse and dependence criteria, and used a diagnostic continuum of severity. Results obtained in the original validation sample of 550 participants showed that the YFAS 2.0 had high internal consistency, and convergent and discriminant validity (high scores on the questionnaire were strongly linked to obesity and binge eating problems) (46). This scale has also been adapted and validated for German, Italian and French populations (48–50).

The food addiction construct, therefore, seems to share clinical characteristics with other addiction types, both substance and behavioral addictions, as well as with eating disorders (45, 51). However, there is a shortage of studies that aim to establish in depth a clear phenotype of these clinical populations, evaluating them together and comparing them with the general population. In order to establish these phenotypes, it is essential to assess food addiction levels in these populations, as well as co-occurring personality traits and psychopathology, given that all these factors are essential in defining and treating these different disorders (52). For this reason, the aim of the current study was to explore the associations of food addiction with clinically relevant variables such as psychopathology, personality, and gender in a sample of patients with an eating disorder (ED), with gambling disorder (GD), and a healthy-weight control group (HC). Additionally, we sought to analyze the psychometric properties of the Spanish version of the YFAS 2.0.

## Materials and methods

### Participants

The sample was recruited between May 2016 and March 2017. The study sample included three groups: a group of *n* = 135 treatment-seeking ED patients, a group of *n* = 166 treatment-seeking GD patients and a HC group of *n* = 197 individuals recruited from the general population. The participants in the ED and GD groups were consecutively recruited from the Eating Disorders Unit at Bellvitge University Hospital in Barcelona, Spain, and the Gambling Disorder Unit at the same hospital. Patients were diagnosed according to DSM-5 criteria (47) by licensed clinical psychologists and psychiatrists. Information regarding the treatment protocols used in the Eating Disorder and Behavioral Addiction Units are explained in Jiménez-Murcia et al. (53) and Fernández-Aranda and Turon (54). Participants in the HC group were recruited from the same university hospital setting to guarantee the equivalence of the geographical origin between study groups. Inclusion criteria for the clinical groups were (1) fulfilling DSM-5 criteria for GD or an ED; and, for all participants, (2) being between 18 and 65 years old. Exclusion criteria for all participants included: the presence of an organic mental disorder, intellectual disability, a neurodegenerative condition, such as Parkinson's disease, or an active psychotic disorder. For the HC group, a lifetime history of ED or GD was an exclusion criterion.

In the ED group, 26 participants met criteria for anorexia nervosa (AN, 19.3%), 43 for bulimia (BN, 31.9%), 29 for binge eating disorder (BED, 21.5%), and 37 for other specified feeding eating disorder (OSFED, 27.4%). In the GD group, 23 patients (13.9%) had mild GD severity (4 or 5 DSM-5 criteria), 51 patients (30.7%) had moderate GD severity (6 to 7 DSM-5 criteria), and 92 subjects (55.4%) had severe GD severity (8 or more DSM-5 criteria).

Table 1 includes a description of the sample (sociodemographic and clinical measures) stratified by group.

**Table 1:** Sample description.

		**Eating disorder patients**		**Gambling disorder patients**		**Healthy control participants**				
		***n** =* **135**		***n** =* **166**		***n** =* **152**				
		***n***	***%***	***n***	***%***	***n***	***%***	**χ^2^**	***df***	***p***
**SEX**										
Females		121	89.6	12	7.2	124	81.6	263.5	2	<0.001
Males		14	10.4	154	92.8	28	18.4			
**EDUCATION LEVEL**										
Primary or less		28	22.2	80	52.6	0	0.0	133.6	4	<0.001
Secondary		87	69.0	59	38.8	148	99.3			
University		11	8.7	13	8.6	1	0.7			
**CIVIL STATUS**										
Single		90	71.4	80	52.6	144	98.0	82.30	4	<0.001
Married or with partner		24	19.0	56	36.8	2	1.4			
Separated or divorced		12	9.5	16	10.5	1	0.7			
**EMPLOYMENT STATUS**										
Unemployed		30	24.6	55	36.2	33	24.8	6.10	2	0.047
Employed		92	75.4	97	63.8	100	75.2			
	α	**Mean**	***SD***	**Mean**	***SD***	**Mean**	***SD***	***F***	***df***	***p***
Age (years-old)		31.35	13.66	40.44	13.11	21.21	3.03	118.5	2/450	<0.001
Body mass index (BMI, kg/m^2^)		26.89	10.17	26.48	4.83	22.12	4.08	22.07	2/450	<0.001
ED severity: EDI-2 total score	0.971	107.91	38.28	−	−	33.60	25.93	368.2	1/285	<0.001
GD severity: total DSM-5 criteria	0.756	−	−	7.46	1.49	−	−	−	−	−
SCL-90-R GSI score	0.982	1.77	0.75	1.19	0.67	0.63	0.46	112.7	2/450	<0.001
TCI-R Novelty seeking	0.776	99.62	16.75	112.56	12.82	99.30	12.16	44.00	2/450	<0.001
TCI-R Harm avoidance	0.892	120.76	18.56	100.22	15.73	98.79	17.89	67.31	2/450	<0.001
TCI-R Reward dependence	0.829	98.13	15.68	99.53	16.44	103.96	14.08	5.59	2/450	0.004
TCI-R Persistence	0.888	106.05	20.99	109.97	19.13	113.86	18.06	5.64	2/450	0.004
TCI-R Self-directedness	0.879	114.20	18.77	127.63	20.75	141.98	16.02	77.16	2/450	<0.001
TCI-R Cooperativeness	0.833	133.62	15.18	128.50	16.61	136.96	14.30	11.62	2/450	<0.001
TCI-R Self-transcendence	0.864	64.76	14.28	62.08	15.16	63.22	13.32	1.22	2/450	0.295

*SD, standard deviation; df, degrees of freedom; α, Cronbach's alpha in the sample; —, The measure is not available for this group*.

### Instruments

#### Yale food addiction scale version 2.0 (YFAS 2.0) (46)

This self-report questionnaire consists of 35 items scored on an eight-level Likert scale (from 0 = never to 7 = every day) and adapted to assess addictive eating behaviors based on DSM-5 SRAD criteria.

The validated Spanish version of the original YFAS demonstrated very good psychometric properties (55): excellent internal consistency for the one single dimension solution (α = 0.95), good accuracy in differentiating between the sample origin (ED vs. controls: specificity equal to 97.6%, sensitivity = 72.8% and area under receiver operating curve AUC = 0.90), good discriminative capacity in screening for specific EDs, and convergent validity compared to external measures of negative affect and depression, general psychopathology, eating disorder severity, and body mass index.

The Spanish YFAS 2.0 includes additional questions that take into account DSM-5 SRAD criteria and follows the scoring guidelines used in the original validation of the YFAS 2.0. These scoring guidelines produces two measurements: (a) a continuous symptom count score that reflects the number of fulfilled diagnostic criteria (ranging from 0 to 11); and (b) a food addiction threshold based on the number of symptoms (at least 2) and self-reported clinically significant impairment or distress. This final measurement allows for the binary classification of food addiction (*present* vs. *absent*). Based on the revised DSM-5 taxonomy, the YFAS 2.0 also provides severity cutoffs for patients who surpass the threshold for food addiction: mild (2–3 symptoms), moderate (4–5 symptoms), and severe (6–11 symptoms).

The YFAS 2.0 was translated into Spanish in accordance with the International Test Commission Guidelines for Translating and Adapting Tests (56). Bilingual clinical psychologists with extensive experience in the ED field translated the original English version into Spanish. This translated Spanish version was then back-translated by a native English speaker and any differences between both versions were discussed and resolved by consensus. The Spanish YFAS Version 2.0 was reviewed by two other Spanish-speaking clinical psychologists, who had not been involved in the back-translation procedure. This was done in order to confirm that the instrument was clear and understandable for younger populations.

#### Eating disorder inventory-2 (EDI-2) (57)

This multidimensional self-report questionnaire includes 91 items to assess cognitive and behavioral characteristics related to eating disorders: drive for thinness, body dissatisfaction, bulimia, ineffectiveness, perfectionism, interpersonal distrust, interoceptive awareness, maturity fears, asceticism, impulse regulation, and social insecurity. A global measure of ED severity can be obtained based on the sum of all the items on the scale. The Spanish validation of this questionnaire obtained excellent psychometrical properties as an external global measure of ED severity (58). Internal consistency for EDI-total scores was excellent in our sample (α = 0.97).

#### Diagnostic questionnaire for gambling disorder according to DSM criteria (59)

This 19-item questionnaire assesses DSM-IV-TR (23) and DSM-5 (47) diagnostic criteria for GD. Convergent validity with external measures of gambling severity in the original validation was very good (*r* = 0.77 for the sample recruited in the general population and *r* = 0.75 for gambling treatment group; (59). Internal consistency of the Spanish version of the questionnaire used in this work was also good (α = 0.81 for the general population and α = 0.77 for gambling treatment samples; (60). Internal consistency for the study was α = 0.76. A global measure of GD severity can be obtained based on the sum of all the items on the scale.

#### Temperament and character inventory-revised (TCI-R) (61)

This self-report questionnaire is designed to evaluate personality traits using 240-items on a five-level Likert scale. It is structured on seven primary personality dimensions: four temperamental factors (novelty seeking, harm avoidance, reward dependence and persistence) and three character dimensions (self-directedness, cooperativeness and self-transcendence). The validated Spanish version used in this study has shown very good internal consistency (Cronbach's alpha α mean value of 0.87) (62). Cronbach's alpha for the TCI-R in the study sample was good to excellent (between α = 0.78 for novelty seeking to α = 0.89 to persistence; see Table 1 for the α-values obtained in each TCI-R scale).

#### Symptom checklist revised (SCL-90-R) (63)

This 90-item self-report questionnaire is widely used for the measurement of perceived psychopathology. It is structured on nine first-order dimensions: somatization, obsessive-compulsive, interpersonal sensitivity, depression, anxiety, hostility, phobic anxiety, paranoid ideation and psychoticism. A global severity index is also used as a global distress index (GSI scale). The Spanish validation of this instrument has shown a mean internal consistency of α = 0.75 (64). In this study, the GSI score was used as an external measure to assess the YFAS 2.0 convergent validity. Cronbach's alpha for this scale in the sample was excellent (α = 0.98).

In addition to the assessment battery, ED and GD patients underwent a semi-structured face-to-face interview to obtain sociodemographic data (age, education level, employment status, and civil status) and other clinical measures (age of disorder onset and disorder duration). This interviewing process has been described previously (53, 54).

### Ethics

This study was carried out in accordance with the latest version of the Declaration of Helsinki. The Ethics Committee of Bellvitge University Hospital (Barcelona, Spain) approved the study, and signed informed consent was obtained from all final participants.

### Data analyses

Data analyses were carried out with Mplus8 (65) and Stata15 (66). Confirmatory Factor Analysis (CFA) assessed the single factor solution for the YFAS 2.0, defining each criterion as categorically dichotomous, and using robust weighted least squared estimator (WLSMV) and delta parameterization. Following previous validation studies for the YFAS, the item measuring impairment-distress was not considered in the CFA since this is a criterion of clinical significance as a whole. Due to the heterogeneity of the sample of the study, the invariance by group (HC, ED, and GD) and sex (male and female) were tested by fitting CFA multi-group models. Goodness-of-fit was considered adequate according to Barrett (67): Root Mean Square Error of Approximation RMSEA < 0.10, Bentler's Comparative Fit Index CFI > 0.90, Tucker-Lewis Index TLI > 0.90, and Weighted Root Mean Square Residual WRMR < 1. Internal consistency was measured through Cronbach's alpha (α, considering α > 0.80 to be adequate).

In this study, different dimensional and categorical measures for the YFAS 2.0 were analyzed. Firstly, the YFAS 2.0 dimensional symptom count, which measures the 11 DSM-5 SRAD criteria (raw scores are in the range of 0–11). And second, the two categorical classifications based on the dimensional symptom count: (a) a threshold for food addition (*present* for individuals with at least 2 symptoms plus self-reported clinically significant impairment or distress, and *absent* for participants who did not meet these criteria); and (b) for patients who met food addiction threshold, a categorical variable classified food addiction severity (*mild* for participants with 2 or 3 symptoms, *moderate* for individuals with 4–5, and *severe* for patients with at least 6 symptoms).

The capacity of the dimensional YFAS 2.0 symptom count to discriminate between the groups was tested through analysis of variance (ANOVA), and the capacity of the YFAS 2.0 categorical classifications to discriminate between the diagnostic subtypes was tested through chi-square tests (χ^2^).

The convergent validity of the YFAS 2.0 with external measures (BMI and EDI-2), personality (TCI-R) and psychopathology scores (SCL-90R GSI) was estimated through Pearson's correlation (*r*, with |*r* |≥ 0.30 considered evidence of a relevant association; (68).

Receiver Operating Characteristics (ROC) methodology assessed the accuracy of the YFAS 2.0 to differentiate between the diagnostic subtypes (ED, GD, and HC). ROC analysis is usually employed in clinical epidemiology and research areas to quantify the accuracy of screening tests and to differentiate between patient states (typically referred to as *diseased* and *non-diseased*) (69). The area under the ROC curve (*AUC*) was estimated as a global measure of the global accuracy-validity of the YFAS 2.0 across all the cutoff points, compared with the external standard of ED group vs. HC group. In these subsamples, the accuracy of food addiction threshold was estimated through the sensitivity (Se) and specificity (Sp) coefficients, and through the Cohen's-kappa measuring the agreement with the external standard of this work (κ, considering κ > 0.40 to be moderate, κ > 0.60 to be good, and κ > 0.80 to be excellent) (70).

Other statistical analysis in the study included χ^2^ procedures to compare the proportions obtained for categorical variables, and ANOVA to compare means obtained for quantitative variables between groups. Estimation of effect size for proportion comparisons and mean comparison was based on Cohen-*d* coefficient, considering |*d*| < 0.20 to be null effect size for, |*d*| > 0.20 to be low effect size, |*d*| > 0.50 to be fair-moderate effect size, and |*d*| > 0.80 to be good-high effect size (68).

## Results

### Food addiction prevalence and symptom count among the groups

**Figure 1:**
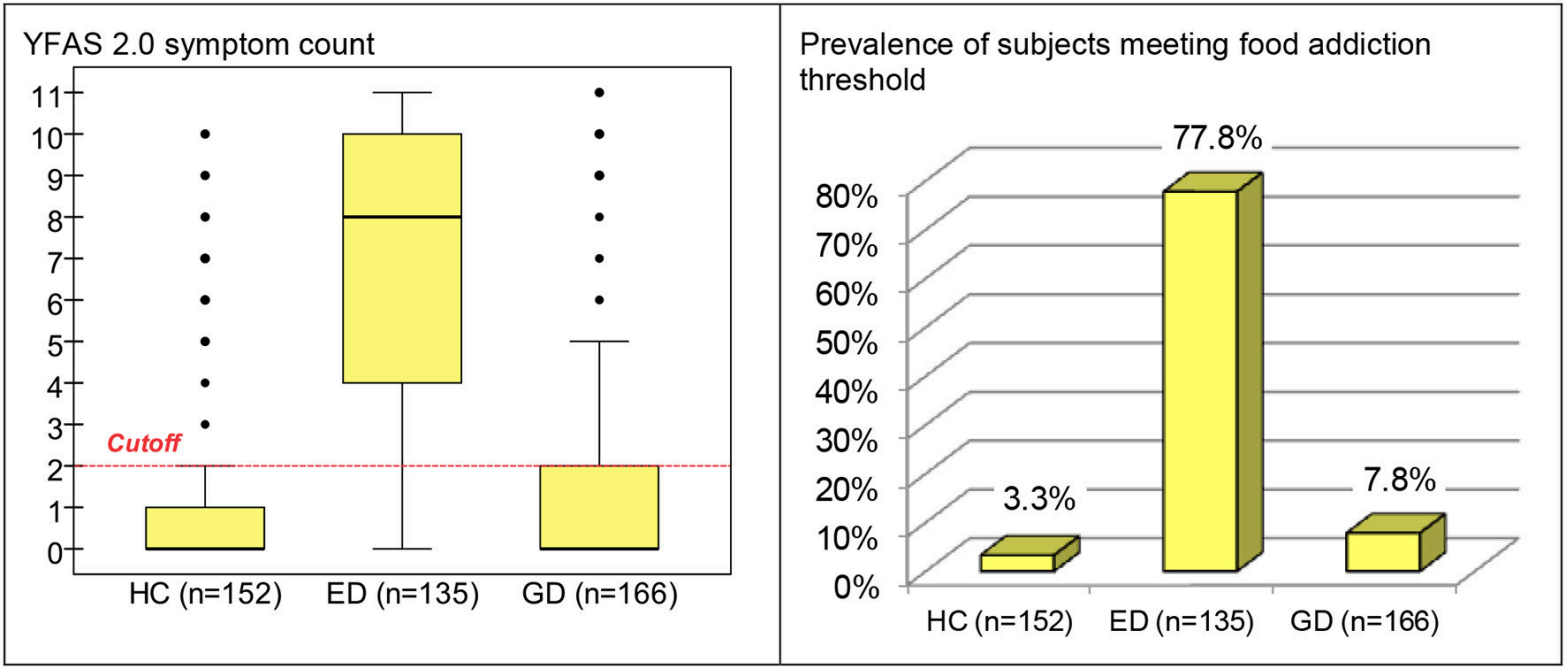
Boxplot for YFAS 2.0 symptom count and bar-chart for prevalence of subjects meeting food addiction threshold (whole sample, stratified by group). HC, healthy control; ED, eating disorder; GD, gambling disorder.

Table 2 includes the distribution of the YFAS 2.0 symptom count in each group and a comparison between them. In the ED group, food addiction symptom count levels were higher in comparison to both GD and HC groups. GD patients obtained higher proportion rates compared to HC for some criteria (although effect sizes were low): consume more than planned, use despite the physical/emotional or interpersonal effects and failure in role obligations. The prevalence of participants who met the threshold for food addiction was statistically equal for HC and GD (3.3 and 7.8%, respectively), and very low compared to the prevalence registered for the ED group (77.8%). Considering the YFAS 2.0 symptom count, the means registered in the three groups were statistically different (0.84 for HC, 1.43 for GD and 6.76 for ED), with the effect size not being relevant when comparing HC with GD patients. Figure 1 contains the box-plots for the symptom count and the bar-charts for those who met the food addiction threshold.

**Table 2:** Capacity of the YFAS 2.0 measures to discriminate between groups

	**ED**		**GD**		**HC**		**ED vs.**		**GD vs.**		**GD vs.**	
	***n = 135***		***n = 166***		***n = 152***		**HC**		**HC**		**ED**	
**YFAS 2.0 criteria**	***n***	***%***	***n***	***%***	***n***	***%***	***p***	***|d|***	***p***	***|d|***	***p***	***|d|***
Consumed more than planned	90	66.7	33	19.9	16	10.5	0.001^*^	**1.41^†^**	0.021^*^	0.26	0.001^*^	**1.07^†^**
Unable to cut down-stop	81	60.0	26	15.7	19	12.5	0.001^*^	**1.14^†^**	0.419	0.09	0.001^*^	**1.03^†^**
Great deal of time spent	87	64.4	27	16.3	24	15.8	0.001^*^	**1.15^†^**	0.908	0.01	0.001^*^	**1.13^†^**
Important activities given up	98	72.6	18	10.8	7	4.6	0.001^*^	**1.95^†^**	0.053	0.24	0.001^*^	**1.61^†^**
Use despite physic-em. effects	90	66.7	20	12.0	8	5.3	0.001^*^	**1.66^†^**	0.039^*^	0.25	0.001^*^	**1.35^†^**
Tolerance	76	56.3	14	8.4	7	4.6	0.001^*^	**1.36^†^**	0.170	0.16	0.001^*^	**1.19^†^**
Withdrawal	91	67.4	24	14.5	18	11.8	0.001^*^	**1.38^†^**	0.491	0.08	0.001^*^	**1.28^†^**
Craving	82	60.7	25	15.1	6	3.9	0.001^*^	**1.53^†^**	0.001^*^	0.39	0.001^*^	**1.07^†^**
Failure in role obligation	75	55.6	15	9.0	2	1.3	0.001^*^	**1.51 ^†^**	0.002^*^	0.35	0.001^*^	**1.15^†^**
Use despite interpers. effects	64	47.4	23	13.9	9	5.9	0.001^*^	**1.06^†^**	**0.009**	0.27	0.001^*^	**0.78^†^**
Use physically hazardous situat.	79	58.5	13	7.8	11	7.2	0.001^*^	**1.30^†^**	0.841	0.02	0.001^*^	**1.28^†^**
Impairment or distress	112	83.0	15	9.0	7	4.6	0.001^*^	**2.58^†^**	0.120	0.18	0.001^*^	**2.21^†^**
**FOOD ADDICTION**												
Positive-present	105	77.8	13	7.8	5	3.3	0.001^*^	**2.33^†^**	0.080	0.20	0.001^*^	**2.00^†^**
**^a^SEVERITY**												
Mild	9	8.6	2	15.4	1	20.0	0.508	0.33	0.814	0.12	0.305	0.21
Moderate	14	13.3	0	0	0	0		**0.55^†^**		0.00		**0.55^†^**
Severe	82	78.1	11	84.6	4	80.0		0.05		0.13		0.17
**YFAS continuous measure**	**Mean**	***SD***	**Mean**	***SD***	**Mean**	***SD***	***P***	**|*d*|**	***P***	**|*d*|**	***P***	**|*d*|**
Food addiction symptoms	6.76	3.75	1.43	2.50	0.84	1.86	0.001^*^	**2.00^†^**	0.049^*^	0.27	0.001^*^	**1.67^†^**

a Classification of severity levels of patients who met the criteria for food addiction.

HC, healthy control; ED, eating disorder; GD, gambling disorder.

* Bold, significant comparison (0.05 level);

† Bold, effect size in the moderate (|d| > 0.50) to good (|d| > 0.80 range).

### Food addiction categories and relevant clinical and personality measures

The first part of Table 3 contains the capacity of the YFAS 2.0 screening threshold to discriminate the clinical measures used in the study, that is, the comparison of the mean clinical scores (BMI, EDI-2 total scores, SCL-90-R, and TCI-R scores) between participants in the food addiction-present vs. absent binary scores. All the means registered in the food addiction = positive/present screening were statistically different than those registered in the food addiction = negative/absent screening, except for in the personality traits novelty seeking, cooperativeness and self-transcendence. Means in the food addiction group were statistically higher for BMI, EDI-2, GSI and harm avoidance, and lower for reward dependence, persistence and self-directedness. The second part of Table 3 contains the means registered in the clinical measures of the study for the groups defined according to YFAS 2.0 food addiction severity levels (mild, moderate and severe). Food addiction with mild severity obtained lower scores than moderate and severe food addiction levels in BMI and the EDI-2 and GSI scales; mild food addiction also registered lower means compared to severe food addiction in harm avoidance and persistence traits.

**Table 3:** Comparison of the YFAS 2.0 screening and severity classifications with clinical measures.

	**Food addiction threshold**						^a^**YFAS 2.0 symptom count**											
	**Absent**		**Present**				**Mild**		**Moderate**		**Severe**		**Mild vs**.		**Mild vs**.		**Moder. vs**.	
	***n*** = **330**		***n*** = **123**				***n*** = **12**		***n*** = **14**		***n*** = **97**		**Moderate**		**Severe**		**Severe**	
	***M***	***SD***	***M***	***SD***	***p***	***|d|***	***M***	***SD***	***M***	***SD***	***M***	***SD***	***p***	***|d|***	***p***	***|d|***	***p***	***|d|***
BMI	24.3	5.7	27.3	9.4	0.001*	0.39	19.1	3.4	24.5	10.9	28.6	9.2	0.168	**0.68**^†^	0.002*	**1.37**^†^	0.165	0.40
^b^EDI-2	41.8	35.2	111.9	36.2	0.001*	**1.96**^†^	77.8	28.7	96.6	46.4	118.0	32.7	0.215	**0.52**^†^	0.001*	**1.31**^†^	0.046*	**0.53**^†^
GSI	0.93	0.64	1.83	0.74	0.001*	**1.30**^†^	1.25	0.34	1.77	0.95	1.91	0.72	0.049*	**0.73**^†^	0.005*	**1.16**^†^	0.538	0.16
NS	104.9	14.7	102.0	16.5	0.081	0.19	99.2	18.2	98.4	19.3	102.8	15.9	0.912	0.04	0.498	0.21	0.393	0.25
HA	100.6	17.8	120.1	18.2	0.001*	**1.08**^†^	111.9	13.8	114.4	27.2	121.8	17.1	0.740	0.12	0.087	**0.64**^†^	0.183	0.33
RD	102.0	15.5	97.0	15.4	0.004*	0.32	101.2	15.1	101.2	14.6	96.0	15.5	0.998	0.00	0.291	0.34	0.273	0.35
PE	111.5	18.9	106.6	20.9	0.022*	0.24	113.8	18.9	115.3	24.9	104.6	20.4	0.869	0.06	0.165	**0.51**^†^	0.049*	**0.51**^†^
SD	134.6	19.7	112.3	18.1	0.001*	**1.18**^†^	118.3	19.1	121.2	20.0	110.4	17.4	0.699	0.15	0.169	0.43	0.050*	**0.57**^†^
CO	133.6	15.7	131.1	16.0	0.151	0.16	136.6	13.7	136.1	15.5	129.8	16.2	0.934	0.04	0.184	0.45	0.204	0.39
ST	62.5	14.4	65.4	13.9	0.064	0.20	66.2	16.2	69.1	18.8	64.8	13.0	0.620	0.17	0.760	0.09	0.323	0.26

a *Classification for patients who met the cutoff for food addiction*.

b *The measure was available for participants in the ED and HC groups*.

BMI, body mass index (kg/m^2^); NS, novelty seeking; EDI-2, EDI-2 total score; GSI, SCL-90R GSI index; HA, harm avoidance; RD, reward dependence; PE, persistence; SD, self-directedness; CO, cooperativeness; ST, self-transcendence; GSI, SCL-90R GSI scale; M, mean; SD, standard deviation. *Bold, significant comparison (0.05 level); †Bold, effect size into the range moderate (|d| > 0.50) to good (|d| > 0.80).

### Associations between the YFAS 2.0 symptom count levels and external measures

Table 4 includes the correlation matrix estimating the associations between the YFAS 2.0 symptom count levels and the participants' age, BMI, and EDI-2 total scores, psychopathology (SCL-90-R GSI scale) and personality traits (TCI-R dimensions). These results indicate that YFAS 2.0 symptom count levels are positively related to BMI, ED severity, psychopathology (GSI index), and harm avoidance. However, a negative correlation between food addiction symptom count levels and self-directedness was found. No correlation between GD severity levels and food addiction severity was found.

**Table 4:** Pearson's correlation between the YFAS 2.0 symptom count with psychological measures.

	**Total**	**ED**	**GD**	**HC**
	***n* = 453**	***n* = 135**	***n* = 166**	***n* = 152**
Age (years-old)	0.121	**0.250^†^**	0.018	0.135
Body mass index (kg/m^2^)	**0.345^†^**	**0.345^†^**	**0.342^†^**	0.098
SCL-90R GSI score	**0.565^†^**	**0.288^†^**	**0.318^†^**	**0.619^†^**
TCI-R Novelty seeking	−0.034	0.189	0.029	0.033
TCI-R Harm avoidance	**0.464^†^**	**0.253^†^**	0.124	**0.280^†^**
TCI-R Reward dependence	−0.183	**−0.247^†^**	−0.057	−0.104
TCI-R Persistence	−0.218	**−0.251^†^**	−0.160	−0.026
TCI-R Self-directedness	**−0.531^†^**	**−0.372^†^**	**−0.334^†^**	**−0.389^†^**
TCI-R Cooperativeness	−0.168	−0.204	**−0.302^†^**	**−0.254^†^**
TCI-R Self-transcendence	0.090	0.064	0.064	0.074
EDI-2 total score		**0.368^†^**	−	**0.658^†^**
GD: total DSM-5 criteria		−	0.060	−

HC, healthy control; ED, eating disorder; GD, gambling disorder; –, The measure is not available for the group.

† Bold, effect size into the range moderate (|r| > 0.24) to good (|r| > 0.30).

### Accuracy of the YFAS 2.0 as a screening/diagnosis tool

The YFAS 2.0 symptom count obtained excellent accuracy in discriminating between HC and the ED group (results in the ROC analysis reported AUC = 0.904). The YFAS 2.0 diagnosis also adequately differentiated between participants in the HC and ED subsamples (Sp = 96.7%, Se = 77.8%, and Cohen's kappa measuring agreement was κ = 0.75).

### Confirmatory factor analysis

Table S1 (supplementary material) contains the complete results for the CFA analysis. The single factor model obtained adequate fit in the whole sample: RMSEA = 0.034, CFI = 0.998, TLI = 0.998, and WRMSR = 0.723. All the items reached very high loadings (above 0.80). The internal reliability coefficient was excellent (α = 0.94). Multi-group analysis did not have a better fit to the data assessing the invariance of the questionnaire structure by group (HC-ED-GD: χ^2^ = 129.04, *df* = 106, *p* = 0.064) and sex (women-men: χ^2^ = 51.73, *df* = 53, *p* = 0.524). These findings suggested that the one dimensional structure was also adequate to represent the structure of the Spanish YFAS 2.0 for male and female, ED, GD, and, HC samples.

Table S2 (supplementary) includes the distribution of the YFAS 2.0 in the study, stratified by the diagnostic subtype and participants' sex. Analyses stratified for each group showed that no statistical differences emerged comparing the means of the YFAS 2.0 symptom count between men and women in the HC group (*F* = 1.12, *df* = 1/150, *p* = 0.293) or in the ED group (*F* = 0.11, *df* = 1/133, *p* = 0.747), but it was when comparing sex in GD (*F* = 4.14, *df* = 1/164, *p* = 0.043).

**Figure 2:**
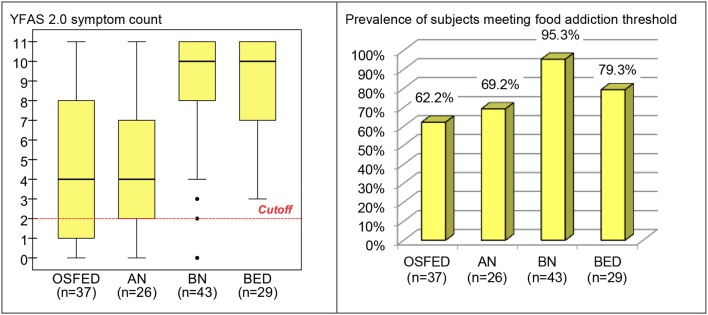
Boxplot for the YFAS 2.0 symptom count and bar-chart for prevalence of subjects meeting food addiction threshold in the (ED subsample, stratified by ED subtype). OSFED, other specified feeding eating disorder; AN, anorexia nervosa; BN, bulimia nervosa; BED, binge eating disorder; ED, eating disorder.

Comparison of the YFAS 2.0 measures between ED subtypes obtained significant results for both the number of reported symptoms (*F* = 20.45, *df* = 3/131, *p* < 0.001) and the presence of food addiction (χ^2^ = 14.04, *df* = 3, *p* = 0.003) (Figure 2). Pairwise comparisons for the YFAS 2.0 symptom count between groups indicated that no difference was present between OSFED and AN (*T* = 0.35, *df* = 131, *p* = 0.732), nor between BN and BED groups (*T* = 0.20, *df* = 131, *p* = 0.839). However, OSFED patients endorsed lower symptom count levels than BN (*T* = 5.77, *df* = 131, *p* < 0.001) and BED patients (*T* = 5.41, *df* = 131, *p* < 0.001); whereas AN obtained lower symptom count levels than BN (*T* = 5.56, *df* = 131, *p* < 0.001) and BED patients (*T* = 5.30, *df* = 131, *p* < 0.001).

## Discussion

There has been growing scientific and clinical interest on whether parallels between the symptomatology present in individuals with substance abuse disorders and excessive food intake can be made (71). The food addiction construct has received increased clinical interest in recent years, although is not recognized as a DSM-5 condition (72, 73). The YFAS 2.0 is the only self-report questionnaire assessing food addiction, referring to addictive-like eating, based on updated DSM-5 SRAD criteria (46). This tool has been validated in different languages, such as in German (48), Italian (50), and French (49). However, this is the first validation of a Spanish language version of the YFAS 2.0 that has been carried out to date. This study confirmed the sound psychometric properties of the Spanish YFAS 2.0. Additionally, we set out to assess the relevance of clinical measures and food-addiction-related variables in a large sample including patients who met criteria for ED or GD, as well as a HC group.

In terms of prevalence, 3.3% of the participants of the HC group met criteria for the presence of food addiction. This finding is lower than the prevalence reported in the general population using previous versions of the YFAS (27) and in the development of this scale (46), but with similar values reported in previous version of the YFAS (2.4% in HC) (55). Taking GD into account, 7.8% of the patients met criteria for the presence of food addiction. This prevalence is in accordance with a recent study obtaining similar values using the original YFAS (19). Finally, regarding ED samples, food addiction prevalence levels were comparable to those reported using the previous version of the YFAS (78% of the ED participants in our study met the food addiction threshold; (18, 55). Additionally, the results of the current study indicate that the YFAS 2.0 has the discriminative capacity to differentiate between ED subtypes (lower food addiction levels were found in OSFED and AN compared to BN and BED), which is also consistent with empirical data reported in previous studies (24, 55). In fact, in our study, the highest percentage of food addiction corresponded to BN group (24, 74). One can postulate that BN and BED patients endorsed higher food addiction severity levels due to the subjective feeling of loss of control and distress that are characterisitic bingeing episodes (75). The extent to which food addiction symptoms might be a premorbid condition of ED symptomatology, or just a consequent factor, could be not be explored with the present research design (51).

As in other studies, we found that higher scores in food addiction symptomatology were positively associated with BMI in ED and GD groups (24). We also found that food addiction prevalence was associated with higher BMI in subjects from the general population (50, 76). However, it must be stressed that not all obese or overweight individuals meet the criteria for “food addiction.” For example, a study in a sample of obese adolescents found that just 38% of the sample met the threshold for food addiction (8). The prevalence found in our adult sample was very similar to that found in other studies (77). A recent meta-analysis also indicated that overweight/obese females aged over 35 years may be more predisposed to food addiction, as assessed by the YFAS (18). While some authors support the food addiction concept by arguing that it shares clinical and neurological traits with addictive disorders (46, 78), others state that it merely serves as an indicator of overeating severity (6).

Numerous similarities between ED and GD have been shown in terms of personality traits and neuropsychological factors (39, 79, 80). Moreover, both disorders have been associated with the presence of food addiction. However, in the present study, food addiction symptom count levels were higher in the ED group compared to both GD and HC groups. Likewise, the prevalence of food addiction was significantly higher in the ED group in comparison with GD and HC groups. Although several studies have postulated that food addiction should be defined as a behavioral addiction due to the striking similarities between overeating and other behavioral addictions like GD (81, 82), our findings suggest that food addiction is strongly associated with ED pathology. However, it is important to bear in mind the gender differences in the clinical groups featured in this study; although GD is a condition that mostly affects males (83), when we consider only women with this condition, the rates of food addiction increase significantly (19).

The YFAS 2.0 threshold also demonstrated good capacity to discriminate between the clinical measures employed in the study. Overall, BMI and psychopathology levels were higher in the group with food addiction compared to those not food addiction meeting criteria. Specifically, participants who met the threshold for food addiction showed significantly higher scores in the GSI scale, indicating elevated general psychopathology levels. Significant differences were also found regarding some personality traits, participants who presented food addiction were characterized by higher levels in harm avoidance and lower self-directedness. These results are in the line with previous food addiction literature by our group indicating that individuals with food addiction, regardless of diagnoses tend to have lower levels of self-directedness (19, 84).

The findings of this study could be used to identify those individuals who best fit the food addiction model. However, more research is required to determine the efficacy of pharmacological and psychological approaches in individuals with food addiction. For example, naltrexone and bupropion have been used for chronic weight management in some obese adults, and, given that these medications are used in the treatment of other substance addictions, it could of interest to know whether patients who report more addictive-like symptoms respond differently (85). Psychological treatment could also have a positive impact on the cognitive processes involved in improving food addiction symptomatology.

### Limitations

The findings of this study must be considered in the context of its limitations. First, the different groups were unbalanced in terms of sex and age. Future studies should aim to include more balanced samples and a control group that is more representative in terms of sociodemographical factors with respect to the clinical groups. Second, food addiction was assessed using a self-report measure which restricts the evaluation of other factors that may be interfering with the obtained results. Third, clinical groups are only constituted by treatment-seeking patients. Therefore, this population is not representative of all individuals with these problematic behaviors. Finally, in the present validation individuals without an ED but with overweight/obesity are underrepresented. Future food addiction studies should attempt to include subjects that reflect the general population.

## Author contributions

GM-B, TS, FF-A, RG, JM, AG, NM-B, ZA, CV-A, ML-M, CS-M, CD, and SJ-M designed the experiment based on previous results and the clinical experience of NA, MG-P, NR, IS, and FC. RG, GM-B, TS, FF-A, MN-A, and SJ-M conducted the experiment, analyzed the data, and wrote a first draft of the manuscript. SJ-M, TS, GM-B, RG, AG, CV-A, ML-M, and FF-A further modified the manuscript.

### Conflict of interest statement

The authors declare that the research was conducted in the absence of any commercial or financial relationships that could be construed as a potential conflict of interest.
