# Corrigendum: Validation of the Spanish Version of the Yale Food Addiction Scale 2.0 (YFAS 2.0) and Clinical Correlates in a Sample of Eating Disorder, Gambling Disorder and Healthy Control Participants

**DOI:** 10.3389/fpsyt.2018.00321

**Published:** 2018-07-12

**Authors:** Roser Granero, Susana Jiménez-Murcia, Ashley N. Gearhardt, Zaida Agüera, Neus Aymamí, Mónica Gómez-Peña, María Lozano-Madrid, Núria Mallorquí-Bagué, Gemma Mestre-Bach, Maria I. Neto-Antao, Nadine Riesco, Isabel Sánchez, Trevor Steward, Carles Soriano-Mas, Cristina Vintró-Alcaraz, José M. Menchón, Felipe F. Casanueva, Carlos Diéguez, Fernando Fernández-Aranda

**Affiliations:** ^1^Ciber Fisiopatología Obesidad y Nutrición, Instituto Salud Carlos III, Barcelona, Spain; ^2^Departament de Psicobiologia i Metodologia, Universitat Autònoma de Barcelona, Barcelona, Spain; ^3^Department of Psychiatry, University Hospital of Bellvitge-IDIBELL, Barcelona, Spain; ^4^Department of Clinical Sciences, School of Medicine and Health Sciences, University of Barcelona, Barcelona, Spain; ^5^Department of Psychology, University of Michigan, Ann Arbor, MI, United States; ^6^CIBER Salud Mental, Instituto Salud Carlos III, Barcelona, Spain; ^7^Laboratory of Molecular and Cellular Endocrinology, Research Area, Complejo Hospitalario Universitario de Santiago de Compostela, A Coruña, Spain; ^8^Department of Physiology, CIMUS, University of Santiago de Compostela-Instituto de Investigación Sanitaria, Santiago de Compostela, Spain

**Keywords:** eating disorders, food addiction, gambling disorder, psychometric properties, validation, YFAS 2.0

An author name was incorrectly spelled as **“Ashley N. Gerhardt**.” The correct spelling is **“Ashley N. Gearhardt**.” The authors apologize for this error and state that this does not change the scientific conclusions of the article in any way. The original article has been updated.

## Conflict of interest statement

The authors declare that the research was conducted in the absence of any commercial or financial relationships that could be construed as a potential conflict of interest.

