# Supplementary material for: Validation of the Spanish Version of the Yale Food Addiction Scale 2.0 (YFAS 2.0) and Clinical Correlates in a Sample of Eating Disorder, Gambling Disorder, and Healthy Control Participants
Source: Front Psychiatry. 2018 May 25;9:208. doi: 10.3389/fpsyt.2018.00208 (PMC5980980; doi:10.3389/fpsyt.2018.00208)
Supplement: Supplementary file 1 [file Table_1.docx]

*Table S1. Results of the Confirmatory Factor Analysis*

|  |  | Multi-group analysis measuring invariance | | | | |
| --- | --- | --- | --- | --- | --- | --- |
|  | Total | Diagnostic subtype | | | Sex | |
|  | sample | ED | GD | HC | Female | Male |
| DSM-5 SRAD criteria | *n=453* | *n=135* | *n=166* | *n=152* | *n=257* | *n=196* |
| Standardized coefficients |  |  |  |  |  |  |
| Consumed more than planned | .926 | .903 | .820 | .884 | .948 | .852 |
| Unable to cut down-stop | .910 | .885 | .845 | .886 | .917 | .883 |
| Great deal of time spent | .890 | .864 | .782 | .870 | .918 | .786 |
| Important activities given up | .893 | .634 | .883 | .710 | .861 | .925 |
| Use despite physic-emot. Effects | .902 | .832 | .714 | .891 | .924 | .807 |
| Tolerance | .947 | .903 | .901 | .849 | .944 | .929 |
| Withdrawal | .904 | .719 | .897 | .935 | .898 | .888 |
| Craving | .900 | .768 | .870 | .835 | .897 | .894 |
| Failure in role obligation | .939 | .852 | .881 | .838 | .936 | .927 |
| Use despite interpersonal effects | .827 | .721 | .801 | .663 | .831 | .850 |
| Use physically hazardous situations | .951 | .909 | .898 | .957 | .950 | .936 |
| Consistency: Cronbach’s alpha (α) | .939 | .899 | .881 | .860 | .943 | .911 |
| Goodness-of-fit |  |  |  |  |  |  |
| Root mean square error approx. (RMSEA) | .034 | .051 |  |  | .032 |  |
| Comparative fit index (CFI) | .998 | .984 |  |  | .998 |  |
| Tucker-Lewis index (TLI) | .998 | .982 |  |  | .998 |  |
| Weighted root mean square resid. (WRMSR) | .723 | .995 |  |  | .992 |  |
| Invariance: joint test |  |  |  |  |  |  |
| Chi-square test |  | 129.04 |  |  | 51.73 |  |
| Degrees of freedom |  | 106 |  |  | 53 |  |
| p-value |  | .064 |  |  | .524 |  |

*Note.* SRAD: substance related and addictive diagnosis.

HC: healthy control. ED: eating disorder. GD: gambling disorder.

Invariance: comparison of the multi-group models with the initial model.

RMSEA: root mean square error of approximation. CFI: comparative fit index. TLI: Tucker-Lewis index.

WRMR: weighted root mean square residual.
