# Supplementary material for: Validation of the Spanish Version of the Yale Food Addiction Scale 2.0 (YFAS 2.0) and Clinical Correlates in a Sample of Eating Disorder, Gambling Disorder, and Healthy Control Participants
Source: Front Psychiatry. 2018 May 25;9:208. doi: 10.3389/fpsyt.2018.00208 (PMC5980980; doi:10.3389/fpsyt.2018.00208)
Supplement: Supplementary file 2 [file Table_2.docx]

*Table S2. Distribution of the YFAS 2.0 in the study*

|  | Eating disorder | | | | Gambling disorder | | | | Healthy control | | | |
| --- | --- | --- | --- | --- | --- | --- | --- | --- | --- | --- | --- | --- |
|  | Women | | Men | | Women | | Men | | Women | | Men | |
|  | *n=121* | | *n=14* | | *n=12* | | *n=154* | | *n=124* | | *n=28* | |
|  | *n* | *%* | *n* | *%* | *n* | *%* | *n* | *%* | *n* | *%* | *n* | *%* |
| *DSM-5 SRAD criteria* |  |  |  |  |  |  |  |  |  |  |  |  |
| Consumed more than planned | 80 | 66.1% | 10 | 71.4% | 3 | 25.0% | 30 | 19.5% | 15 | 12.1% | 1 | 3.6% |
| Unable to cut down-stop | 69 | 57.0% | 12 | 85.7% | 5 | 41.7% | 21 | 13.6% | 16 | 12.9% | 3 | 10.7% |
| Great deal of time spent | 78 | 64.5% | 9 | 64.3% | 3 | 25.0% | 24 | 15.6% | 19 | 15.3% | 5 | 17.9% |
| Important activities given up | 89 | 73.6% | 9 | 64.3% | 1 | 8.3% | 17 | 11.0% | 6 | 4.8% | 1 | 3.6% |
| Use despite physic-emot. effects | 81 | 66.9% | 9 | 64.3% | 2 | 16.7% | 18 | 11.7% | 8 | 6.5% | 0 | 0.0% |
| Tolerance | 67 | 55.4% | 9 | 64.3% | 3 | 25.0% | 11 | 7.1% | 6 | 4.8% | 1 | 3.6% |
| Withdrawal | 84 | 69.4% | 7 | 50.0% | 5 | 41.7% | 19 | 12.3% | 17 | 13.7% | 1 | 3.6% |
| Craving | 72 | 59.5% | 10 | 71.4% | 4 | 33.3% | 21 | 13.6% | 6 | 4.8% | 0 | 0.0% |
| Failure in role obligation | 67 | 55.4% | 8 | 57.1% | 3 | 25.0% | 12 | 7.8% | 2 | 1.6% | 0 | 0.0% |
| Use despite interpers. effects | 56 | 46.3% | 8 | 57.1% | 2 | 16.7% | 21 | 13.6% | 8 | 6.5% | 1 | 3.6% |
| Use physically hazardous sit. | 71 | 58.7% | 8 | 57.1% | 3 | 25.0% | 10 | 6.5% | 10 | 8.1% | 1 | 3.6% |
| Impairment or distress | 101 | 83.5% | 11 | 78.6% | 3 | 25.0% | 12 | 7.8% | 6 | 4.8% | 1 | 3.6% |
| *Food addiction* Present | 94 | 77.7% | 11 | 78.6% | 3 | 25.0% | 10 | 6.5% | 5 | 4.0% | 0 | 0% |
| *^1^ Severity* Mild | 8 | 8.5% | 1 | 9.1% | 0 | 0% | 2 | 20.0% | 1 | 20.0% | 0 | 0% |
| Moderate | 14 | 14.9% | 0 | 0% | 0 | 0% | 0 | 0% | 0 | 0% | 0 | 0% |
| Severe | 72 | 76.6% | 10 | 90.9% | 3 | 100% | 8 | 80.0% | 4 | 80.0% | 0 | 0% |
| *YFAS continuous raw score* | ED-Women | | ED-Men | | GD-Women | | GD-Men | | HC-Women | | HC-Men | |
| Minimum | 11 | | 0 | | 0 | | 0 | | 0 | | 0 | |
| Maximum | 11 | | 11 | | 11 | | 11 | | 10 | | 7 | |
| Mean | 6.73 | | 7.07 | | 2.83 | | 1.32 | | 0.91 | | 0.50 | |
| Standard deviation | 3.76 | | 3.85 | | 3.76 | | 2.35 | | 1.96 | | 1.35 | |
| Percentiles P_25_ | 4 | | 5 | | 0 | | 0 | | 0 | | 0 | |
| P_50_ | 8 | | 7 | | 1 | | 0 | | 0 | | 0 | |
| P_75_ | 10 | | 11 | | 6 | | 2 | | 1 | | 1 | |

*Note.* ^1^Distribution for patients who met the cutoff for food addiction screening/diagnosis present.

SRAD: substance related and addictive diagnosis.

HC: healthy control. ED: eating disorder. GD: gambling disorder.
